# Compressive Strength of Newly Developed Nonsintered Hydroxyapatite Blocks for Bone Graft Applications

**DOI:** 10.1055/s-0043-1774327

**Published:** 2023-11-23

**Authors:** Sunarso Sunarso, Abdi Suryadi, Decky Joesiana Indrani, Azizah Intan Pangesty

**Affiliations:** 1 Departement of Dental Materials Science, Faculty of Dentistry, Universitas Indonesia, Jakarta, Indonesia; 2 Department of Dental Materials Science, Faculty of Dentistry, Universitas Indonesia, Jakarta, Indonesia; 3 Department of Metallurgical and Materials Engineering, Faculty of Engineering, Universitas Indonesia, UI Campus, Depok, West Java, Indonesia

**Keywords:** nonsintered, hydroxyapatite, block, hydrothermal, compressive strength

## Abstract

**Objective** This study aimed to fabricate and evaluate the phase purity and compressive strength of the nonsintered hydroxyapatite (HA) block obtained via phase transformation of set calcium sulfate dihydrate (CSD) block under hydrothermal conditions at different temperatures.

**Materials and Methods** Nonsintered HA block was prepared by immersion CSD block (4 mm in diameter and 8 mm in height) in a 1 mol/L sodium phosphate (Na _3_ PO _4_ ) solution under hydrothermal conditions at 100°C, 140°C, and 180°C for 48 hours. X-ray diffraction was used to determine the crystalline phase of the obtained blocks. The mechanical strength of the blocks was measured using a compressive strength test.

**Results** The result shows that the CSD block could be fully transformed into a HA block at 180°C for 48 hours without changing its macroscopic shape. The compressive strength of the obtained blocks was lower compared with the CSD block.

**Conclusion** The current method has successfully produced a nonsintered HA block at 180°C for 48 hours. The compressive strength of the HA block decreased compared with the gypsum block used as a precursor. However, the compressive strength of the HA block that was produced still falls within the range of cancellous bone.

## Introduction

Hydroxyapatite (HA) is known as a biocompatible and osteoconductive bone graft material. 1 It has been used for decades with good clinical outcomes. 2 Despite good clinical results, its slow resorption is the main drawback of HA bone grafts. 3 4 Commercial HA bone grafts are often prepared through sintering. This type of HA has low resorption during implantation. Nonsintered HA bone graft would be more resorbable compared with sintered one. 5

Deproteinized bovine bone is one of the most widely used nonsintered HA. 6 7 8 Bio-Oss is one of the deproteinized bovine bone products that is used clinically. It is derived from the bovine bone using a chemical reaction to remove organic substances. However, deproteinized bovine bone HA such as Bio-Oss also has slow resorption. 4 Tadjoedin et al 9 have reported that the resorption rate of Bio-Oss is 10% per year. Another study reported that Bio-Oss has remained even after 4.5 years of implantation. Several studies have been reported on the fabrication of nonsintered HA other than the deproteination method. One method was developed by Suzuki et al to produce an HA block using calcium sulfate dihydrate (CSD) as a precursor via the dissolution–precipitation technique. 10 The results suggested that HA was formed. However, brushite was also found in the fabricated block as impurities.

Previously, we successfully fabricated new nonsintered HA blocks through the phase transformation of gypsum blocks under hydrothermal conditions. 11 The gypsum block with a diameter of 6mm and height of 3mm could be fully transformed into an HA block at 180°C for 24 hours. However, the compressive strength of the HA block has not been known. Therefore, this research aimed to evaluate the compressive strength of the newly developed non-sintered HA block.

## Materials and Methods

### Sample Preparation

Nonsintered HA blocks were prepared according to the method that was previously reported with the modification of sample dimension and hydrothermal reaction time. Calcium sulfate hemihydrate (CSH) was mixed with distilled water at the liquid-to-CSH powder ratio of 0.5 according to the previous report. 11 The paste was molded in a cylindrical split mold (4 mm in diameter and 8 mm in height) made of acrylic. Briefly, the paste was taken from the mixing bowl using a spatula and placed in the mold. Two glass slides were put at the top and the bottom of the mold and pressed with paper clips to firm it. The paste was left to set for 24 hours at room temperature. The set gypsum blocks were immersed in a polytetrafluoroethylene (PTFE)-lined vessel containing 1 mol/L sodium phosphate (Na _3_ PO _4_ ).12H _2_ O (Merck, Darmstadt, Germany) solution. The PTFE-lined vessel was then placed in a hydrothermal vessel consisting of a shell made of stainless steel. The hydrothermal vessel was put in an oven at 100°C, 140°C, and 180°C for 48 hours. After hydrothermal, the specimens were washed with distilled water three times and dried at 37°C for 24 hours.

### Material Characterization

The gypsum block and the obtained blocks were crushed into a powder. The powders were characterized using X-ray diffraction (XRD) (PANanalytical-Xpert Pro; CuKα, λ = 1.54Å). The XRD characterization was performed at a current of 30 mA and voltage of 40 kV with the step size of 0.0170 from 2θ of 10.0084 to 89.9764°. Rietveld refinement of the obtained XRD peaks was employed using Xpert Highscore software to determine the phase composition, lattice parameter, and crystallite size of the HA crystal formed in each specimen. An automatic Rietveld profile was used (Pseudo-Voigt fitting).

### Mechanical Test

For mechanical strength measurement, the gypsum and the obtained blocks were subjected to compressive strength tests using a universal testing machine (Shimadzu, AGSX-50Kn). The load cell was 500 N with a crosshead speed of 0.5 mm/minute. The force at which the block started to break was recorded. Nine specimens were used for each group to determine their compressive strength. The number of specimens was calculated by the Federer formula. The average value of compressive strength was then calculated.

### Data Analysis

Statistical analysis was done to determine the significance of the mechanical strength among the samples using SPSS Software. Shapiro–Wilk test was used to determine the normality of the data, followed by one-way analysis of variance. The significance of the compressive strength among the group of samples was calculated by Tamhane post-hoc analysis, in which a *p* -value of 0.05 was considered significant.

## Results

### Characterization of HA Blocks

**Fig. 1:**
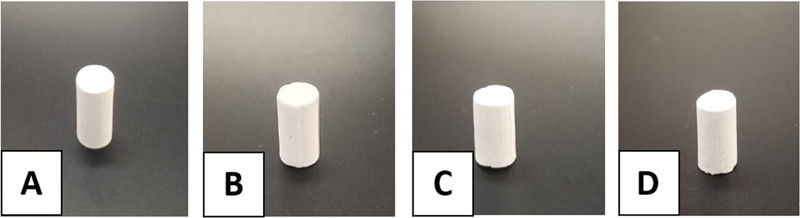
Photograph of gypsum block ( **A** ) and the obtained block after immersion in sodium phosphate (Na _3_ PO _4_ ) at 100°C ( **B** ), 140°C ( **C** ), and 180°C ( **D** ) for 48 hours. CaSO4, calcium sulphate.

**Fig. 2:**
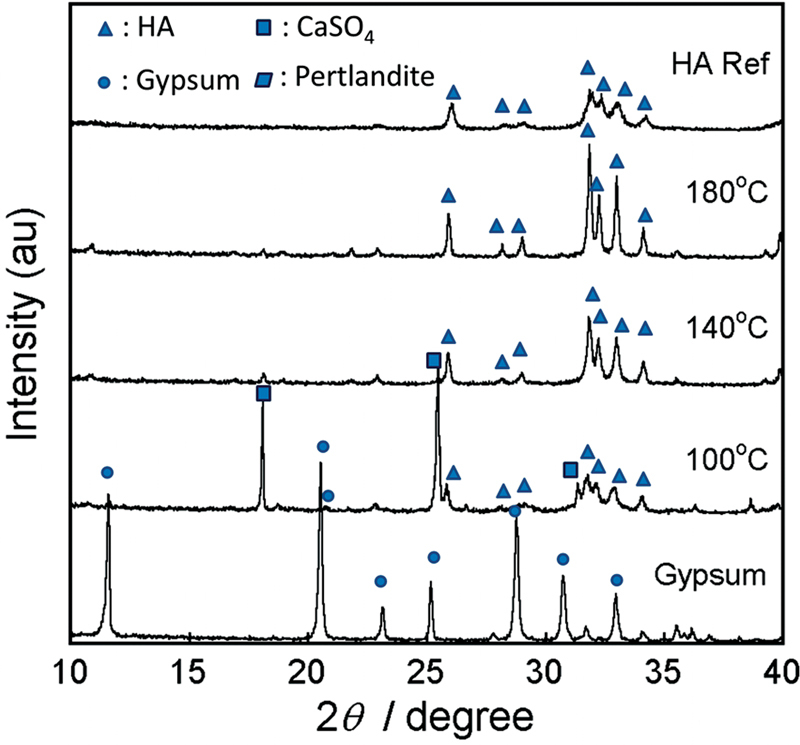
X-ray diffraction peaks of gypsum block, the obtained block after immersion in sodium phosphate (Na _3_ PO _4_ ) at 100°C, 140°C, and 180°C, and hydroxyapatite reference.

Fig. 1 shows the photograph of the gypsum block and the obtained block after hydrothermal. After hydrothermal, the block did not crumble or collapse. Fig. 2 demonstrated the XRD peaks of gypsum and the obtained blocks. The XRD peaks were indexed using a crystallography open database (COD). After immersion at 100°C, the gypsum phase was transformed into HA (COD: 96–230–0274). However, calcium sulphate (CaSO _4_ ) anhydrate (COD: 96–900–4097), gypsum (COD: 96–901–3165), and portlandite (COD: 96–900–0114) phases were also found as listed in Table 1 . As the temperature increased to 140°C, the obtained block was composed of 97.5% HA and 2.5% portlandite (Ca(OH) _2_ ). At the temperature of 180°C, the obtained block is considered fully transformed HA (99.5%) with only a trace amount of portlandite (0.5%). The intensity of HA peaks was higher with the increasing temperature. Crystallite size increased with the increasing temperatures ( Table 2 ). The *a* -lattice decreased with the increase in temperature. The *c* -lattice increased from 100°C to140°C but decreased at 180°C ( Table 2 ).

**Table 1:** Crystal phases of the obtained block after immersion in 1 mol/L Na _3_ PO _4_ at 100°C, 140°C, and 180°C for 48 hours

Group	Phases
HAP-100	Hydroxyapatite (63.6%) CaSO _4_ anhydrate (28.2%) Gypsum (4.2%) Portlandite (4.0%)
HAP-140	Hydroxyapatite (97.5%) Portlandite (2.5%)
HAP-180	Hydroxyapatite (99.5%) Portlandite (0.5%)

Abbreviations: CaSO4, calcium sulphate; Na _3_ PO _4_ , sodium phosphate.

**Table 2:** Unit cell parameter and crystallite size of the obtained blocks obtained using Rietveld analysis

Sample name	Lattice parameter			Crystallite size (nm)
	*a (Å)*	*b (Å)*	*c (Å)*	
HAP-100	9.433	9.4335	6.8906	20.66
HAP-140	9.422	9.4226	6.8929	34.86
HAP-180	9.418	9.4187	6.8869	65.06

### Compressive Strength

**Fig. 3:**
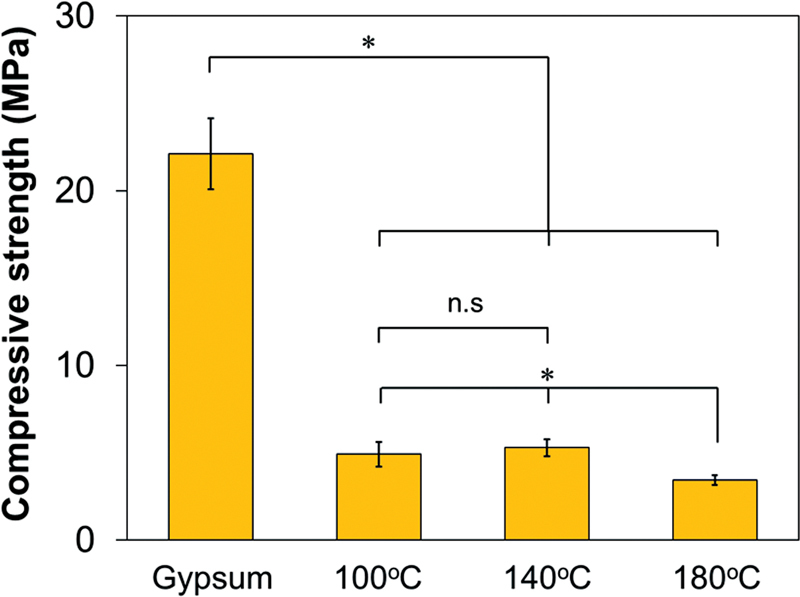
Compressive strength of gypsum block and the obtained block after immersion in sodium phosphate (Na _3_ PO _4_ ) at 100°C, 140°C, and 180°C ( *n* : 6; * *p* < 0.05, n.s.: not significant).

The compressive strength of the gypsum and the obtained blocks were shown in Fig. 3 . Gypsum block showed a compressive strength value of 22.11 ± 2.03 MPa. After immersion in Na _3_ PO _4_ solution, the compressive strength was decreased to 4.92 ± 0.70 MPa, 5.28 ± 0.49 MPa, and 3.43 ± 0.27 MPa for HAP-100, HAP-140, and HAP-180, respectively ( Table 3 ). The compressive values from each specimen per group were subjected to the Shapiro–Wilk normality test. Normal distribution was obtained from the test. Further, the homogeneity test was performed and the data showed not homogenous. Thus, Tamhane's post-hoc analysis was done to see the difference between the group. The difference in compressive strength value between the gypsum block and the obtained blocks after hydrothermal was statistically significant. However, within the obtained blocks, only HAP-180 shows a significant value compared with both HAP-100 and HAP-140. Meanwhile, between HAP-100 and HAP-140, the values were not significant.

**Table 3:** List of average compressive strength of gypsum block and the obtained block after immersion in Na _3_ PO _4_ at 100°C, 140°C, and 180°C compared with human cancellous bone

Group	Average compressive strength (MPa)
Gypsum	22.11 ± 2.03
100°C	4.92 ± 0.70
140°C	5.28 ± 0.49
180°C	3.43 ± 0.27
Cancellous bone	0.1–16

Abbreviation: Na _3_ PO _4_ , sodium phosphate.

## Discussion

HA has been widely used as a bone graft in dentistry. 12 13 14 15 Bovine-based HA is among the most applied bone grafts besides sintered HA due to their biocompatibility and osteoconductivity. 16 17 18 19 The main drawback of bovine HA and sintered HA is their slow resorption during implantation. This study attempted to fabricate nonsintered HA to improve resorption. Previously, the newly developed nonsintered HA showed better solubility compared with sintered HA in an acetate buffer solution that simulates osteoclastic environments. 10 The solubility of bone graft material in acetate buffer was reported to directly correlate to its resorption during implantation. 20 21 Higher solubility was thought due to the carbonate content found in the HA crystal. It was reported that carbonated HA showed higher solubility in osteoclastic simulation due to the release of carbonate ions. 21

This study aimed to evaluate the compressive strength of nonsintered HA obtained via phase transformation of gypsum block under hydrothermal conditions. Besides, phase purity, lattice parameter, and crystallite size of the obtained blocks were also evaluated. In this study, a nonsintered HA block could be fabricated via hydrothermal reaction using a gypsum block. The obtained blocks were not collapsed after hydrothermal treatment, which suggests the method preserved the original sample shape. This is important since it could be used for other complex shapes.

Based on the XRD, hydrothermal reaction at 100°C produced not only the HA phase but also the CaSO _4_ anhydrate, gypsum, and portlandite phases. CaSO _4_ anhydrate and portlandite were most probably intermediate phases that formed before they transformed completely into the HA phase. The gypsum phase was still detected at 100°C (4.2%). At 140°C, CaSO _4_ anhydrate and gypsum phases were transformed completely into the HA phase, and only 2.5% portlandite remained. The gypsum phase was no longer detected at 140°C. Further increase in hydrothermal temperature to 180°C produced HA blocks with the highest purity (99.5%) and only a trace amount of portlandite phase (0.5%) was detected. Therefore, the gypsum block could be considered fully transformed into an HA block. It is known that HA is the most stable calcium phosphate phase at alkaline pH. 22 In this study, gypsum block was immersed in Na _3_ PO _4_ solution that has a very basic pH; thus it would create a condition for HA precipitation. As a result, an HA block could be formed. The lattice parameters of the obtained blocks were changed with the increasing hydrothermal temperature. Our previous results suggest that these changes might be due to the crystal growth and the substitution of carbonate ions into the HA crystal.

Phase transformation from gypsum block into HA block decreased compressive strength considerably. Based on Fig. 3 , the decrease in compressive strength was up to 75% regardless of the reaction temperatures. The lowest compressive strength value was shown in HAP-180 where the gypsum block was considered fully converted to HA phase. The decrease in compressive strength might be caused by the change in microstructure due to the phase transformation of the gypsum block into HA. Previously, our research group has found that after phase transformation, more pores were formed between the interlocked crystals of the HA block compared with that of the gypsum block precursor. 10 These more pores observed in the obtained blocks might cause a decrease in compressive strength.

The HA block obtained in this study is intended for nonload-bearing applications. Nevertheless, obtaining an HA block having compressive strength close to that of human bone is preferred. It was reported that the compressive strength of human cancellous bone is ranged between 0.1 and 16 MPa. 23 In this study, the compressive strength of the obtained blocks decreased after phase transformation due to the formation of HA crystals. The decrease in compressive strength is expected not to affect the material's performance clinically. The obtained HA-180 has a compressive strength of 3.43 MPa, which is still in the range of human cancellous bone.

Although the nonsintered HA block has shown promising results as a bone graft candidate with better resorbability than bovine bone, additional evaluation is necessary before it can be used in clinical settings. Further evaluations include cytotoxicity and animal tests to prove the osteoconductivity of the material.

## Conclusion

Gypsum block could be fully transformed into HA block via hydrothermal reaction at 180°C for 48 hours. The compressive strength of the obtained blocks decreased significantly compared with the gypsum block with the increase in the HA phase. The compressive strength of the obtained HA block is still in the range of that of cancellous bone.

## References

[1] Kumar P Vinitha B Fathima G Bone grafts in dentistry J Pharm Bioallied Sci 2013 5 01 S125 S127 23946565 10.4103/0975-7406.113312 PMC3722694

[2] Capello W N D'Antonio J A Manley M T Feinberg J R Hydroxyapatite in total hip arthroplasty. Clinical results and critical issues Clin Orthop Relat Res 1998 355 200 211 9917605

[3] Orlovskii V P Komlev V S Barinov S M Hydroxyapatite and hydroxyapatite-based ceramics Inorg Mater 2002 38 10 973 984

[4] Schorn L Fienitz T De Donno F Critical-size defect augmentation using sintered and non-sintered bovine bone matrix - an experimental controlled study in minipigs J Oral Maxillofac Surg 2021 79 09 1866 1873 34051155 10.1016/j.joms.2021.03.025

[5] Ayukawa Y Suzuki Y Tsuru K Koyano K Ishikawa K Histological comparison in rats between carbonate apatite fabricated from gypsum and sintered hydroxyapatite on bone remodeling BioMed Res Int 2015 2015 579541 26504813 10.1155/2015/579541 PMC4609359

[6] Narukawa M Suzuki O Mayahara M Resorption analysis of deproteinized cancellous bovine bone Dent Mater J 2020 39 05 760 765 32404567 10.4012/dmj.2019-240

[7] Hämmerle C H Chiantella G C Karring T Lang N P The effect of a deproteinized bovine bone mineral on bone regeneration around titanium dental implants Clin Oral Implants Res 1998 9 03 151 162 10530129 10.1034/j.1600-0501.1998.090302.x

[8] Baldini N De Sanctis M Ferrari M Deproteinized bovine bone in periodontal and implant surgery Dent Mater 2011 27 01 61 70 21112618 10.1016/j.dental.2010.10.017

[9] Tadjoedin E S de Lange G L Bronckers A L Lyaruu D M Burger E H Deproteinized cancellous bovine bone (Bio-Oss) as bone substitute for sinus floor elevation. A retrospective, histomorphometrical study of five cases J Clin Periodontol 2003 30 03 261 270 12631185 10.1034/j.1600-051x.2003.01099.x

[10] Suzuki Y Matsuya S Udoh K Fabrication of hydroxyapatite block from gypsum block based on (NH4)2HPO4 treatment Dent Mater J 2005 24 04 515 521 16445012 10.4012/dmj.24.515

[11] Sunarso R Qalbina T Indrani D J Herda E Pangesty A I Effect of hydrothermal temperature on phase transformation and mechanical property of non-sintered hydroxyapatite and its in vitro solubility J Korean Ceram Soc. 2023 60 01 215 223

[12] Pepla E Besharat L K Palaia G Tenore G Migliau G Nano-hydroxyapatite and its applications in preventive, restorative and regenerative dentistry: a review of literature Ann Stomatol (Roma) 2014 5 03 108 114 25506416 PMC4252862

[13] Balhuc S Campian R Labunet A Negucioiu M Buduru S Kui A Dental applications of systems based on hydroxyapatite nanoparticles—an evidence-based update Crystals (Basel) 2021 11 06 674

[14] Mazumder S Nayak A K Ara T J Hasnain M S Hydroxyapatite composites for dentistryApplications of Nanocomposite Materials in Dentistry United Kingdom Woodhead Publishing 2019 123 143

[15] Izzetti R Gennai S Nisi M Gulia F Miceli M Giuca M R Clinical applications of nano-hydroxyapatite in dentistry Appl Sci (Basel) 2022 12 21 10762

[16] Kim R W Kim J H Moon S Y Effect of hydroxyapatite on critical-sized defect Maxillofac Plast Reconstr Surg 2016 38 01 26 27441185 10.1186/s40902-016-0072-2 PMC4932121

[17] Shaikh M S Husain S Lone M A Lone M A Akhlaq H Zafar M S Clinical effectiveness of anorganic bovine-derived hydroxyapatite matrix/cell-binding peptide grafts for regeneration of periodontal defects: a systematic review and meta-analysis Regen Med 2020 15 12 2379 2395 33356535 10.2217/rme-2020-0113

[18] Antoun H Eid J Zouiten O Histologic and histomorphometric analysis at 26 months of a bovine hydroxyapatite maxillary sinus graft: a case report Int J Periodont Restor Dent 2018 38 04 557 563 10.11607/prd.2727 29077774

[19] Zubaidah N Kurnati S Febrianti N N Nurdianto A R Oktaria W Luthfi M The pattern of osteocyte in dental socket bone regenerative induced by hydroxyapatite bovine tooth graft Bali Med J 2022 11 03 1489 1493

[20] van Gestel N AP Schuiringa G H Hennissen J HPH Resorption of the calcium phosphate layer on S53P4 bioactive glass by osteoclasts J Mater Sci Mater Med 2019 30 08 94 31414232 10.1007/s10856-019-6295-x PMC6694093

[21] Ishikawa K Miyamoto Y Tsuchiya A Hayashi K Tsuru K Ohe G Physical and histological comparison of hydroxyapatite, carbonate apatite, and β-tricalcium phosphate bone substitutes Materials (Basel) 2018 11 10 1993 30332751 10.3390/ma11101993 PMC6213161

[22] Ishikawa K Bone substitute fabrication based on dissolution-precipitation reactions Materials (Basel) 2010 3 02 1138 1155

[23] Gerhardt L C Boccaccini A R Bioactive glass and glass-ceramic scaffolds for bone tissue engineering Materials (Basel) 2010 3 07 3867 3910 28883315 10.3390/ma3073867 PMC5445790

